# Use of Therapeutic Plasma Exchange and Intravenous Immunoglobulin to Prevent Complications in a K+ Sensitized Pregnancy

**DOI:** 10.7759/cureus.72254

**Published:** 2024-10-24

**Authors:** Mary D Hanson, Darren Groh, Michael Barsoom

**Affiliations:** 1 Pathology, Creighton University School of Medicine, Omaha, USA; 2 Maternal/Fetal Medicine, Creighton University School of Medicine, Omaha, USA

**Keywords:** fetal anemia, hemolytic disease of the fetus and newborn, kell, plasmapheresis, therapeutic plasma exchange, transfusion medicine

## Abstract

The K antigen is a major cause of hemolytic disease of the fetus and newborn (HDFN). K-HDFN is unique in that it can result in destruction of not just mature erythrocytes but fetal erythrocyte progenitors, causing severe fetal anemia earlier in pregnancy than other antigens. This poses a danger to fetal health as intrauterine transfusion (IUT), the preferred method of managing HDFN, becomes riskier earlier in pregnancy. This report follows a K-negative mother managed with an alternative treatment, designed to delay the need for IUT.

The patient is a 32-year-old K-negative female, G2P1001, sensitized against K by her previous pregnancy with a 256 anti-K antibody titer. To prevent HDFN, she opted for preventative treatment to lower her immune response. She received three rounds of therapeutic plasma exchange, which lowered her titer to 64, followed by weekly IVIG administration at a dosage of 1g/kg body weight. Fetal anemia was monitored via middle cerebral artery Doppler imaging.

The fetus did require two IUTs; however, they were not required until the third trimester. A healthy baby was delivered at 36 weeks with mild anemia and a positive direct antiglobulin test.

The standard of care for K-sensitized pregnancies involves watchful waiting and correction of anemia with IUT. However, K-HDFN, if untreated, can cause severe anemia in early pregnancy when IUT is less viable. This case study joins a handful of others in reported literature where a K-sensitized pregnancy was treated prophylactically with immune-modulating therapies and argues that these treatments deserve further recognition and study.

## Introduction

Hemolytic disease of the fetus and newborn (HDFN) is a severe and potentially fatal condition caused when a mother’s immune system becomes sensitized against her fetus’s red blood cell (RBC) antigens. Once sensitization has occurred, the goal of therapy becomes only to minimize complications [1]. The current standard of care is to monitor fetal anemia non-invasively via repetitive ultrasound readings of middle cerebral artery peak systolic velocity, which increases to more than 1.5 multiples of median (MoM) in HDFN [2]. Once anemia is suspected, the next steps are more invasive: cordocentesis followed by intrauterine transfusion (IUT) [2-4]. In the hands of a capable practitioner, IUT is typically a safe procedure [5,6]. However, certain conditions make IUT more dangerous, such as early second-trimester pregnancy (before 18-22 weeks) [1,2,7-9], or hydrops fetalis, a late consequence of HDFN [7]. 

While the risk posed by early IUT is a concern, the procedure has been largely successful in protecting alloimmunized pregnancies from fetal loss due to HDFN and has been broadly accepted as a good treatment for the condition [1]. However, IUT alone may not be the only, or best, treatment for all types of alloimmunizations. Over 50 different antigens are known to cause HDFN [2], and not all of them behave in the same way. While not the most common antigen, K (also called Kell or KEL1) is the third most immunogenic human RBC antigen and the second most major cause of HDFN after RhD, with an approximate prevalence in the general population of .0001-.006% (compared to RhD-HDFN at ~0.5%) [1,10,11]. The K antigen possesses unique qualities that can pose a challenge to clinicians attempting to care for a patient with a K-sensitized pregnancy.

Most immunogenic RBC antigens are present in mature fetal RBCs. Their targeting and subsequent destruction by maternal antibodies lead to fetal anemia with a high reticulocyte count. However, K is present in fetal erythrocyte precursors as early as week ten of development [7], leading to the destruction of not just mature RBCs but developing cells as well [7,10,12,13]. This can have serious consequences in a K-sensitized pregnancy, where fetal anemia can present earlier in the second trimester than in HDFN caused by other antibodies, leading to serious HDFN and hydrops fetalis before the time when IUT can be employed as a viable treatment [7,11,14].

This leads to the question of whether more can be done in pregnancies where alloimmunization has already occurred to decrease the maternal immune response and prevent HDFN. Two possible avenues of treatment include therapeutic plasma exchange (TPE) and Intravenous Immunoglobulin (IVIG) administration. While there have been relatively few studies examining these treatments, due to the low prevalence of the K antigen, limited case reports and retrospective studies have demonstrated a correlation between TPE/IVIG and improved outcomes. These outcomes include improved survival rates, higher fetal hemoglobin, increased time before IUT was required, and decreased necessity of IUT as compared to control groups or previous pregnancies [1,2,7-9,12,15]. While these treatments can be given independently of each other, the largest amount of data is available for combined TPE/IVIG pregnancies [1].

In this case, we present a K-negative patient who was sensitized against the K antigen by a previous pregnancy. In an effort to prevent fetal loss, she has been treated with IVIG and TPE in a regimen similar to many of the cited case studies.

## Case presentation

The patient is a 32-year-old white female, G2P1001 with one healthy child born seven years prior at the same hospital, presenting with anti-K antibodies for the first time at the maternal antibody screen. Though most patients acquire alloimmunization through transfusion with K+ blood [16], this patient had no previous transfusion history. Instead, she likely became sensitized through her previous uneventful pregnancy with her K+ husband. The mother was typed as O+, K-, and an eluate was performed to ensure that the only antibodies initially present in the mother’s blood were anti-K. Given this information, it was determined that the fetus was at risk for K-HDFN.

**Figure 1:**
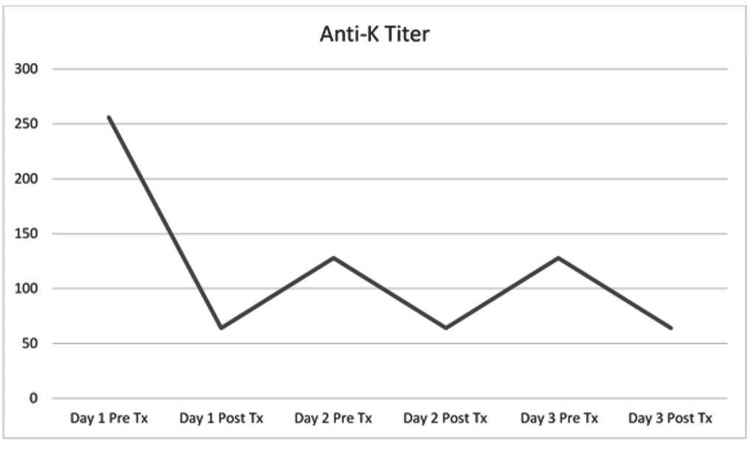
Anti-K titer levels, measured before and after therapeutic plasma exchange (TPE) treatments Pre Tx: Pre-treatment; Post Tx: Post-treatment

After the risks of HDFN were discussed with the patient, the family decided to attempt combination treatment with IVIG and TPE. In a treatment plan modeled after a previous successful case series, three plasma exchanges were performed over the course of gestational week 13, with one treatment every other day. Using the Spectra Optia system and a central venous catheter, approximately 3L of patient plasma were removed each session and replaced by 2L of Octapharma 5% albumin and 1L plasma; 3g calcium gluconate was also infused to prevent citrate-mediated hypocalcemia. No adverse events were reported. Before and after each procedure, the patient’s anti-K titers were measured using the Ortho Vision gel testing system, which performed an indirect antiglobulin test of the patient’s plasma against K+ red blood cells in serial dilutions. The titer, which began at 256, trended downward but leveled off at 64 (Figure 1). 

After completing plasma exchange, the patient was scheduled for weekly IVIG administration at a dosage of 1g/kg body weight from week 14 until delivery. From week 15 onwards, she was followed with MCA doppler to assess for fetal anemia. Due to increased MCA velocity in weeks 31 and 34 (up to 1.52 MoM), two IUTs were performed during the pregnancy. 109 mL of 77% HCT and 150 mL of 78% HCT irradiated PRBCs were transfused, respectively, with no complications. Percutaneous uterine blood sampling taken during the procedure confirmed the fetus’s K+ status via molecular testing using the Precise Type Human Erythrocyte Antigen 1.2 BeadChip DNA array. The array found that the fetus was K+k+, consistent with the family history.

The original plan of care involved induction of labor at 36 weeks; however, the fetus was found to be in malposition. Therefore, an elective Cesarean section was performed at 36 weeks, 2 days, and the patient gave birth to a male infant with Apgar scores of 8 at one minute and 9 at five minutes.

After birth, the infant was found to have a positive direct antiglobulin test (DAT), low reticulocyte count, and decreased RBC count (see Table 1) consistent with K-antibody-mediated destruction of erythrocyte precursors. However, the anemia was not severe and the infant did not require post-birth transfusion. 

**Table 1:** Infant laboratory results post-birth DAT: Direct Antiglobulin Test

Test	Result	Reference Range
RBC	4.4	4.7-6.3 m/uL
Hemoglobin	13.4	13.0-23.0 g/dL
Hematocrit	41.30%	45-65%
MCV	94	92-120 fl
Reticulocyte	13.2	150-410 k/uL
Bilirubin	1.7	1.1-10.5 mg/dL
DAT	1+	0

## Discussion

While it is impossible to say what the outcome of this pregnancy would have been without treatment, this report represents a case where combined TPE/IVIG may have positively influenced the course of an alloimmunized pregnancy. Two intrauterine transfusions were required to maintain fetal hemostasis; however, neither were emergent and both occurred well into the third trimester. The danger of K-mediated HDFN lies not just with the need for IUT but with the possibility of fetal anemia in the early second or even first trimester, before the point when IUT is a safe and viable treatment for anemia. With the combined TPE/IVIG treatments, we hoped to avoid early fetal anemia and early IUT, and in this case, that outcome was successful. 

The treatment of alloimmunization in pregnancy, particularly involving the K antigen, is controversial. Current recommendations involve close monitoring of fetal anemia with Doppler, IUT, and early delivery [16]. TPE and IVIG treatments individually remain Category III recommendations for alloimmunization during pregnancy, based on low-quality evidence supporting their use [17-19]. Controlled trials, which would raise the quality of evidence and potentially raise the recommendation level, are difficult to obtain due to the uncommon nature of the K antigen [7,20]. However, a handful of small case studies have demonstrated a correlation between TPE and IVIG treatments and improved outcomes in the case of K-immunized pregnancies [1,2,7-9,12,15,20,21]. These case studies are important, as they show clinicians struggling with K-immunized pregnancies alternative treatment options and also help provide grounds for insurance coverage for patients seeking this treatment [7]. The treatment regimen in this case is most similar to a small case series of five women, where three rounds of TPE administered in weeks 10-13 of pregnancy successfully decreased antibody titers before weekly IVIG treatments began a few days later culminating in healthy deliveries [9]. This is comparable to treatment protocols that have already been employed at this hospital in the past for anti-D sensitized pregnancies, with three rounds of TPE at week 7 followed by weekly IVIG [22]. This method in turn had been modified from previous case reports which utilized IVIG only up to 20 weeks gestation or more TPE sessions [12]. However, the core principles of early TPE to decrease antibody titer followed immediately afterward by IVIG to maintain lowered immune response remain the same.

While TPE and IVIG treatments are not well-studied in K alloimmunized pregnancies [12], their mechanisms of action are well known. TPE reduces inappropriate immune response by physically siphoning off plasma, which contains the maternal antibodies [2,8,12]. This can cause a rebound effect later on, which IVIG has been shown to reduce [8,12].

IVIG consists of pooled antibodies from thousands of donors [22]. It competitively blocks the Fc receptors of the placenta, preventing maternal anti-K antibodies from entering fetal circulation [7,8,12,21]. It may also induce an Fc blockade in the fetal reticuloendothelial system, preventing fetal macrophages from phagocytizing antibody-marked RBCs [7,8,12,21,23]. Finally, it is believed to cause increased maternal antibody turnover, helping to remove pathogenic anti-K antibodies from circulation [12,21,23]. This treatment, combined with the earlier TPE, can help reduce the maternal immune response to a K+ fetus.

## Conclusions

This case report details the pregnancy of a K-sensitized mother and a K+ fetus that was prophylactically treated with immune-modulating therapy to prevent a need for early IUT. The pregnancy progressed well with IUT needed only in the third trimester and the delivery of a healthy K+ infant. It is possible that this pregnancy may have been viable without the TPE/IVIG therapy, and it is also possible that without this therapy, the pregnancy would have ended in severe early anemia and fetal death. K-sensitization is unpredictable and dangerous in pregnancy, and the combined TPE/IVIG treatment regimen offers a potential way for clinicians to protect their pregnant patients from the uncertainty of HDFN. More data on TPE/IVIG therapy for K alloimmunization is required to form a consensus, but this report joins many other small case studies that show a correlation between TPE/IVIG combination therapy and satisfactory outcomes. 
